# Mean initial cerebral saturation and time to start advanced life support in out-of-hospital cardiac arrest: are they correlated?

**DOI:** 10.1186/cc13680

**Published:** 2014-03-17

**Authors:** C Genbrugge, C De Deyne, I Meex, F Jans, W Boer, J Dens

**Affiliations:** 1 ZOL, Genk, Belgium

## Introduction

During advanced life support (ALS) it is still impossible to predict return of spontaneous circulation (ROSC) or outcome. Cerebral saturation (rSO_2_) measurements with near-infrared spectroscopy can be used in cardiac arrest circumstances and could play a role in predicting ROSC or neurologic outcome [1]. It is known that an initial rhythm of asystole and a long no/low-flow time has a worse outcome. We measured rSO_2 _during ALS in out-of hospital cardiac arrest (OHCA) patients and compared the mean rSO_2 _during the first minute with the time between the emergency call (EC) and start of ALS.

## Methods

With IRB approval, rSO_2 _was prospectively measured between December 2011 and November 2013 in 51 OHCA cases with presumed cardiac cause during ALS. One sensor of the EQUANOX Advance was applied to the right side of the patient's forehead when the medical emergency team arrived. The measurement was continued until death of the patient or arrival at the ICU. Survivors (S) are defined as sustained ROSC longer than 20 minutes. CPR data were collected using the Utstein CPR data registration. The Mann-Whitney test and Pearson chi- square were utilized for comparison of S and nonsurvivors (NS) data. The Pearson test was used to examine correlation.

## Results

Of the 51 patients, 21 were S. Mean age was 70 years (± 15) in the S, of which 10 (48%) were male, and in the nonsurvivors (NS) mean age was 70 years (± 17) (*P *= 0.916) with 23 (77%) male patients (*P *= 0.042). The initial rhythm was asystole in 11 S and in 20 NS (*P *= 0.386), pulseless electrical activity in two S and NS (*P *= 1), and ventricular fibrillation in eight S and six NS *(P *= 0.207). The arrest was witnessed in 15 NS and 16 S (*P *= 0.083). Lay rescuer BLS was performed in 17 NS and nine S (*P *= 0.4). A significant difference in time of EC and start of ALS was observed between S (12 minutes; 8 to 15) and NS (14 minutes; 12 to 17) (*P *= 0.03). Mean initial rSO_2 _was 34% (± 23) and 24% (± 12.5) in S and NS (*P *= 0.077). We observed a negative correlation between mean initial rSO_2 _and time between EC and ALS (correlation coefficient -0.243; *P *= 0.041).

## Conclusion

A tendency towards higher mean initial rSO_2 _in S compared with NS was observed together with a negative correlation between mean initial rSO_2 _and time between the EC and start of ALS. Also a significant difference in time between the EC and start of ALS between S and NS is observed. Larger studies are needed to confirm the possible function of rSO_2 _as a surrogate for time between the EC and start of ALS.
